# Curcumin induces G2/M arrest and triggers autophagy, ROS generation and cell senescence in cervical cancer cells

**DOI:** 10.7150/jca.45176

**Published:** 2020-09-25

**Authors:** Tuan Wang, Xia Wu, Mus'ab Al rudaisat, Yinjing Song, Hao Cheng

**Affiliations:** Department of Dermatology and Venereology, Sir Run Run Shaw Hospital, School of Medicine, Zhejiang University, Hangzhou, 3 Qingchun Road, Zhejiang, 310016, P.R. China.

**Keywords:** curcumin, autophagy, ROS, cell senescence

## Abstract

Our study explored the tumor-suppressive effect of curcumin on cervical cancer cells. Cervical cancer is one of the most common cancers among women worldwide. Acquired resistance to chemotherapeutics and toxicity of such drugs has undermined the effectiveness of cervical cancer treatments. Therefore, the identification of novel chemotherapeutics is key to improving the survival of patients with cervical cancer. Curcumin has been shown to have various bioactivities, including antioxidant and antitumor effects; however, its effect on cervical cancer remains elusive. Here, we used the SiHa human cervical cancer cell line to test various concentrations of curcumin on the proliferation and apoptosis of cervical cancer cells. The involvement of autophagy and reactive oxygen species (ROS) in these effects were also tested by using specific autophagy inhibitors and ROS scavengers. Our results showed that curcumin induced ROS accumulation, apoptosis, autophagy, cell cycle arrest, and cellular senescence accompanied by upregulation of p53 and p21 proteins in SiHa cells.

## Introduction

Cervical cancer is one of the most common cancers among women worldwide. In recent years, acquired resistance of tumor cells to chemotherapeutics and toxicity of these drugs, have undermined the effectiveness of cervical cancer treatments 1. The available vaccines against human papillomavirus (HPV) infections cannot completely prevent cervical cancer or demonstrate any therapeutic effect. In addition, it is infeasible to control cervical cancer or precancerous lesions through vaccines 2. Therefore, development of novel therapeutic drugs is the key to improve chemotherapy success rate alongside the survival of patients and their life quality 3.

Reactive oxygen species (ROS) are active compounds that generally contain oxygen and result from aerobic metabolism 4. In the past, ROS were generally thought to be merely toxic by-products of aerobic metabolism. However, recent studies have indicated that ROS can act as signaling molecules and play crucial roles in diverse signal transduction pathways 5,6. Autophagy is a recycling system whereby cells degrade cytoplasmic proteins and organelles through the lysosomal pathway 7. Cells can modulate their activity by adjusting the number of their components via autophagy. The initiation of autophagy is accomplished by a group of specialized genes called autophagy-related genes. Autophagy can protect cells from dying or induce cell death, depending on the circumstances 8. ROS and autophagy-related proteins, which regulate the autophagic response to cellular stress, counterbalance each other via multiple complex signaling pathways 9. Under certain circumstances, ROS can also induce cellular damage and perturb specific signal transduction pathways, thereby leading to autophagic cell death, also termed Type II cell death 10. In addition, ROS are important components of the c-Jun N-trminal kinase (JNK) signaling pathway and thus play vital roles in the inflammatory response and apoptosis. On one hand, ROS are involved in the regulation of the physiological activities of cells; on the other hand, they can also induce oxidative damage to critical organelles and DNA, thereby promoting cellular senescence 11.

Cellular senescence is a programmed response of healthy cells to various stresses. It manifests as permanent cell cycle arrest 12. Molecular degenerative events, such as telomere shortening and DNA damage induce premature senescence *in vitro*, and ROS-induced oxidation of DNA is among the major contributors to DNA damages. Multiple studies have indicated that escape from cellular senescence is one of the key steps of tumorigenesis. Chemoradiotherapy is one of the most effective and widely used approaches in cancer treatment. Given that tumor cells occasionally demonstrate an aging phenotype after chemoradiotherapy, specific induction of cellular senescence may prove an effective therapeutic approach, in addition to apoptosis induction therapy in cancer.

Curcumin is a pigment extracted from the rhizomes of *Curcuma longa*, a perennial herb of the *Zingiberaceae* (ginger) family. Studies have suggested that curcumin exhibits bioactivities, such as anti-(microbial, oxidant, inflammatory, and tumor) and free-radical-scavenging effects 13. It has been shown a substantial inhibition to the transcription of *HPVE6/E7* and the proliferation of cervical cancer cell lines 14. In addition, drugs containing turmeric products can clear virginal HPV infections in mice 3. However, the specific mechanisms underlying these effects are yet to be clarified. Curcumin also exerts significant inhibitory effects during tumor formation and progression. Although there have been studies exploring the involvement of oxidative stress, DNA damage and repair, cell cycle arrest, and apoptosis, the mechanism(s) underlying the tumor-suppressive effects of curcumin remain elusive. We investigated the effect of different dose of curcumin on human cervical cancer Siha cells. We found that curcumin was able to induce cellular senescence in those cells. Moreover, we observed that this process was preceded and accompanied by apoptosis, autophagy, ROS accumulation.

## Methods

### Cell culture

Siha cells were preserved in the dermatology lab of Sir Run Run Shao Hospital. They were maintained in Dulbecco's modified Eagle's medium (DMEM) supplemented with 10% (v/v) heat-inactivated fetal bovine serum (FBS) and 1% penicillin-streptomycin. Cells were cultured at 37 °C in a humidified 5% CO_2_-95% air incubator. Curcumin was dissolved in dimethylsulfoxide (DMSO) at a concentration of 10 mM and was stored in a dark-coloured bottle at -20 °C. The stock was diluted to the required concentration with DMEM when needed. Prior to curcumin treatments, cells were grown to about 80% confluence and then exposed to curcumin at different concentrations (0-50 µM) for different periods of time (0-48 h). Cells grown in medium containing an equivalent amount of DMSO without curcumin served as control.

### Cell proliferation analysis

Cells were grown in 96-well microtiter plates (10000 cells/well) and then incubated for 24 h in the presence of various doses of curcumin (0-50 µM) in the absence or presence of N-Acetyl-L-cysteine (NAC) or Z-Val-Ala-Asp(Ome)-FMK (Z-VAD). At the required time point, the medium was removed and 200 µl CCK-8 (5 mg/ml in medium) was added to each well. The plates were incubated for a further 4 h at 37 °C. After incubation, the medium was removed from all the wells. The coloured solution was quantified at 450 nm using a micro-plate reader (Spectra Max 190; Molecular Devices, Sunnyvale, CA).Cell viability was determined as percent of the control. Each condition was performed in triplicate wells, and data were obtained from at least 3 separate experiments. The results were expressed as the mean values ±SD. Statistical analysis was performed by student's test (Prism). *P* < 0.05 was considered to be significant.

### Detection and quantification of acidic vesicular organelles with acridine orange staining

Autophagy is the process of sequestering cytoplasmic proteins into the lytic component and is characterized by the formation and promotion of acidic vesicular organells (AVOs) as described previously. For detection of the acidic cellular compartment, we used acridine orange, which emits bright red fluorescence in acidic vesicles but fluoresces green in the cytoplasm and nucleus. Cells were seeded in 24 well plates and treated with curcumin for hours. Acridine orange was then added at a final concentration of 1 µg/ml for periods of 15 min. Pictures were obtained with a fluorescence microscope (Axioskop). For autophagy inhibition, cells were pretreated with 20 nM Baf-A1 for 1 h and then incubated with curcumin.

### Monitoring autophagic flux and mCherry-EGFP-LC3 transfection

The siha cells were collected, adjusted to a cell concentration of 5.0×10^4^/ml, seeded in 24-well plates, added with 500 µl of culture medium per well, then cultured at 37 °C in 5% CO_2_ overnight, and the mCherry-EGFP-LC3 plasmid was transferred into siha cells. 24 hours later, with the treatment of 0 µM, 30 µM curcumin, curcumin 30 µM + Baf-A1 20 nM, the distribution of autophagic vesicles was observed under laser confocal microscopy.

### Western blot analysis

Total proteins were scraped into RIPA lysis buffer with protease inhibitors then measured protein concentration by the Bradford Assay Kit (Bio-Rad). Equal amounts of protein were separated electrophoretically in 8% or 12% SDS-polyacrylamide gels and transferred to nitrocellulose membranes. The membranes were incubated with specific antibodies at 4 °C overnight, after washed with TBST for three times, the membranes were detected using appropriate secondary antibodies and ECL reagents as recommended by the manufacturer. The consequences were analyzed via the specialized software.

### Cell death analysis by fluorescence

Propidium iodide (PI) is a kind of nucleic acid dye, it cannot penetrate the intact cell membrane, but due to the increased permeability of cell membranes of dead cells and late apoptotic cells, PI can penetrate the cell membrane to make the nucleus Stained red. Siha cells were plated onto coverslips, treated with indicated curcumin for 24 h and fixed. Cells were incubated with Propidium iodide (PI) then DAPI was used to localize the nucles, and observed under a fluorescence microscope.

### Apoptosis analysis by flow cytometer

Apoptosis induced by treatment with curcumin was assayed using propidium iodide (PI). Cells were incubated with a range of curcumin for 24 h or 48 h before they were harvested by centrifugation. Harvested cells were washed twice with phosphate-buffered saline (PBS), fixed in 70% ethanol (in PBS) on ice overnight and then resuspended in PBS containing 40 µg/ml PI, 0.5 mg/ml RNase and 0.1% Triton X-100. After 30 min at 37 °C in the dark, the cells were analyzed with a flow cytometer (Beckman-coulter Cytomics FC500, San Jose, CA, USA). The results were analyzed using Wincycle software.

### Measure of ROS generation

To determine ROS in curcumin-treated cells, FACS analysis was performed. Cells were treated with a range of curcumin for 24 h and then exposed to 5 mM NAC or Baf-A1 20 nM for 30 min prior to staining. The cells were stained with 5 µg/ml DCF-DA for 30 min, and ROS generation was analyzed using the flow cytometer and fluorescence microscope.datas were analyzed by Cell Quest software (Becton-Dickinson, San Jose, CA).

### Cell cycle analysis

Siha cells were plated at a density of 1.0×10^6^ cells/well into 6-well flat-bottom tissure culture plates and treated with different concentrations of curcumin (30 and 50 µM) or vehicle control DMSO for 24 h, or with Cur 50 µM + NAC. After the treatment, the cells were harvested, washed twice with ice cold PBS (pH 7.4) and fixed in 70% ethanol for overnight at 4 °C. then, the cells were incubated with 250 µl of RNase A (100 µg/ml) for 30 min at 37 °C and the cells were finally stained with 500 µl of propidium iodide (50 µg/ml) for 1 h in the dark. Stained cells were analyzed with BD FACS calibur flow cytometer (BD Biosciences, San Jose, CA). Three independent experiments were performed.

### Detection of senescence associated-beta-Galactosidase

Detection of senescence associated-beta-Galactosidase (SA-beta-gal) was performed according to protocol. Briefly, siha cells were fixed with 2% formaldehyde, 0.2% glutaraldehyde in PBS, then washed and exposed overnight at 37 °C to solution containing 1 mg/ml 5-bromo-4-chloro-3-indolyl-b-o-galactopyranoside, 5mM potassium ferrocyanide, 5 mM potassium ferricyanide, 150 mM Nacl, 2 mM MgCl_2_, and 0.1 M phosphate buffer, pH 6.0 then observed cells under microscope.

### Statistical Analysis

Statistical analysis was performed using Graphpad Prism 5 software. Data difference comparison was performed by two-way anova analysis. When *P*<0.05, it was indicated by *, *P*<0.01 was expressed by **, and statistical data were from more than three independent parallel experiments.

## Results

### ROS accumulation caused by curcumin in Siha cells

As a polyphenol, curcumin can induce abnormal energy metabolism and ROS accumulation in cells. Accordingly, we utilized the 2′,7′-Dichlorofluorescin diacetate (DCF-DA) fluorescence probe to evaluate the changes in intracellular ROS levels after treating SiHa cells with curcumin. DCF-DA is a non-polar compound that can rapidly penetrate the cell membrane and is converted into DCFH by intracellular esterase. Intracellular ROS can oxidize DCFH into the fluorescent DCF, whose intensity is proportional to the amount of intracellular ROS.

**Figure 1:**
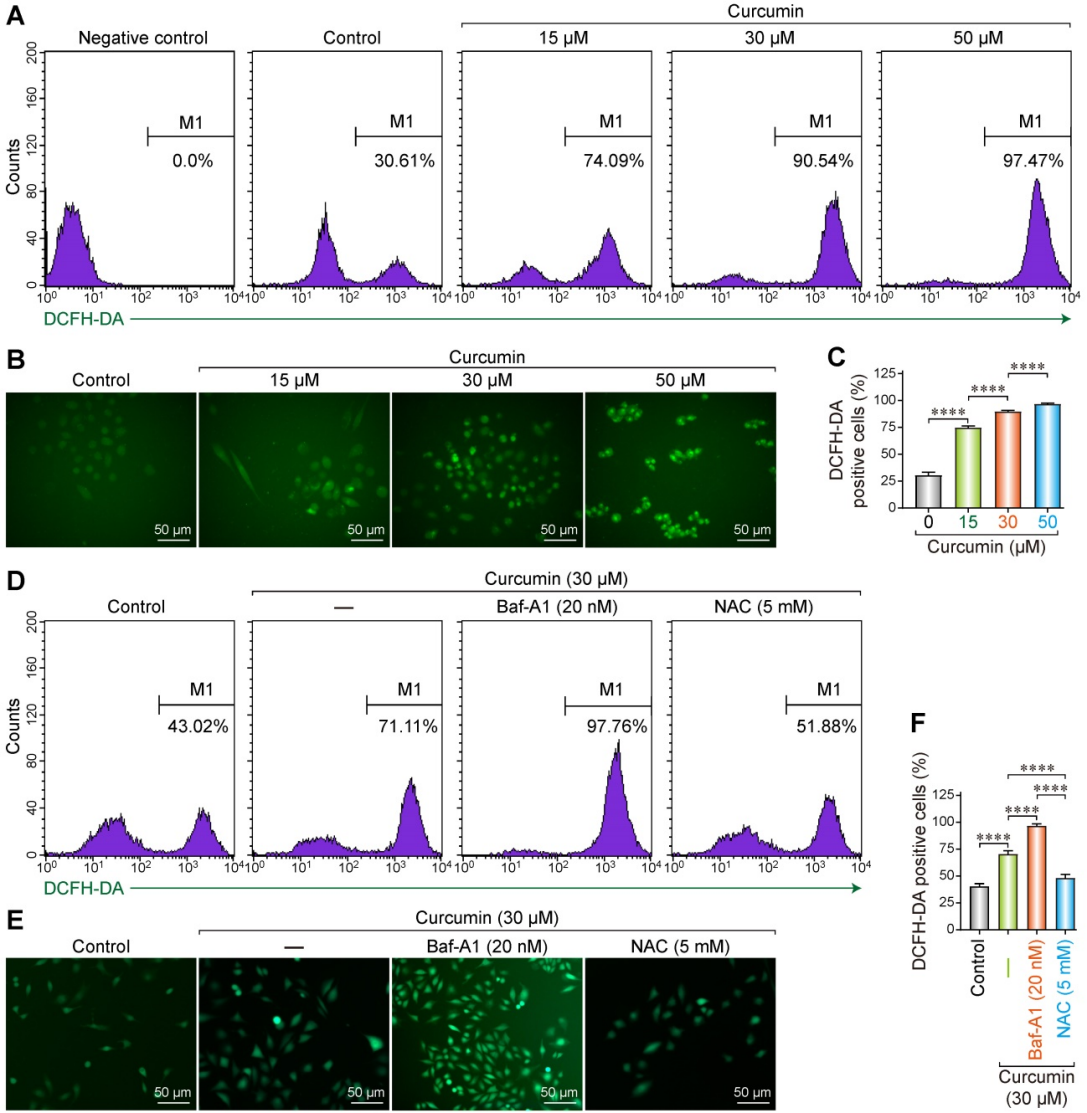
Reactive oxygen species generation in curcumin treated siha cells. **(A)** Siha cells treated with curcumin in a concentration-dependent manner, then flow cytometry was used for detection of fluorescence. Positive cells are given on each image. **(B)** Representative photomicrograph of ROS measurement through DCF-DA. Siha cells were treated with curcumin of different concentrations for 24 h, incubated with DCF-DA (10 µM) for 30min and ROS levels were determined as described under a fluorescent microscope.**(C)** The densitometric measurements of the florescence images were plotted in graph taking Mean and ±SEM and statistical analysis were carried out using one-way ANOVA (*p* < 0.001 vs control).** (D)** Siha cells were treated with curcumin 30 µM for 24 h, Cur combination with Baf-A1 (20 nM), Cur combination with NAC (5 mM), then flow cytometry was used for detection of fluorescence. Positive cells are given on each image.** (E)** Representative photomicrograph of ROS measurement through DCF-DA. **(F)** Significant differences were noted in all comparisons: control versus Cur only, Cur only versus Cur+Baf-A1 (20 nM), Cur only verus Cur+NAC (5 mM).

After DCF-DA fluorescence staining, cells treated with curcumin displayed green fluorescence in a dose-dependent manner. Flow cytometry results showed that the proportion of the fluorescent cells was 30.61% in the negative control, whereas the samples treated with 15, 30, and 50 µmol/L curcumin had 74.09%, 90.54%, and 97.47% fluorescent cells (Figure 1A). All the differences were statistically significant. These results were confirmed with fluorescent microscopy on DCF-DA stained cells, whereby we determined that the proportion of the fluorescent cells was 75%, 90%, and 97% with 15, 30, and 50 µmol/L of curcumin (Figure 1B,C), respectively.

To further analyze the correlation between curcumin-induced ROS accumulation and autophagy, cells were treated with 30 µM of curcumin for 24 h with or without Baf-A1, or NAC. The proportion of the fluorescent cells in the curcumin-treated group was approximately 71.11% compared to that of the negative control. This proportion increased to approximately 97.76% when Baf-A1 was co-administered. NAC co-treatment significantly decreased the proportion of the fluorescent cells to 51.88% (Figure 1D-F). These findings indicate that autophagy facilitates the clearance of intracellular ROS. The inhibition of autophagy upregulates ROS, which can be effectively removed by the antioxidant NAC. The results of fluorescence microscopy were consistent with flow cytometry results.

### Curcumin induced G2/M cell cycle arrest and inhibited cell proliferation

To investigate the effect of curcumin on the proliferation and morphology of SiHa cells, the cells were cultured in media with various concentrations (0, 5, 15, 30, or 50 µmol/L) of curcumin for 24 h, allowing the formation of curcumin + N-Acetyl-L-cysteine (NAC) or + Z-Val-Ala-Asp(Ome)-FMK (Z-VAD) groups. We then measured the cell survival rates using the CCK-8 method.

**Figure 2:**
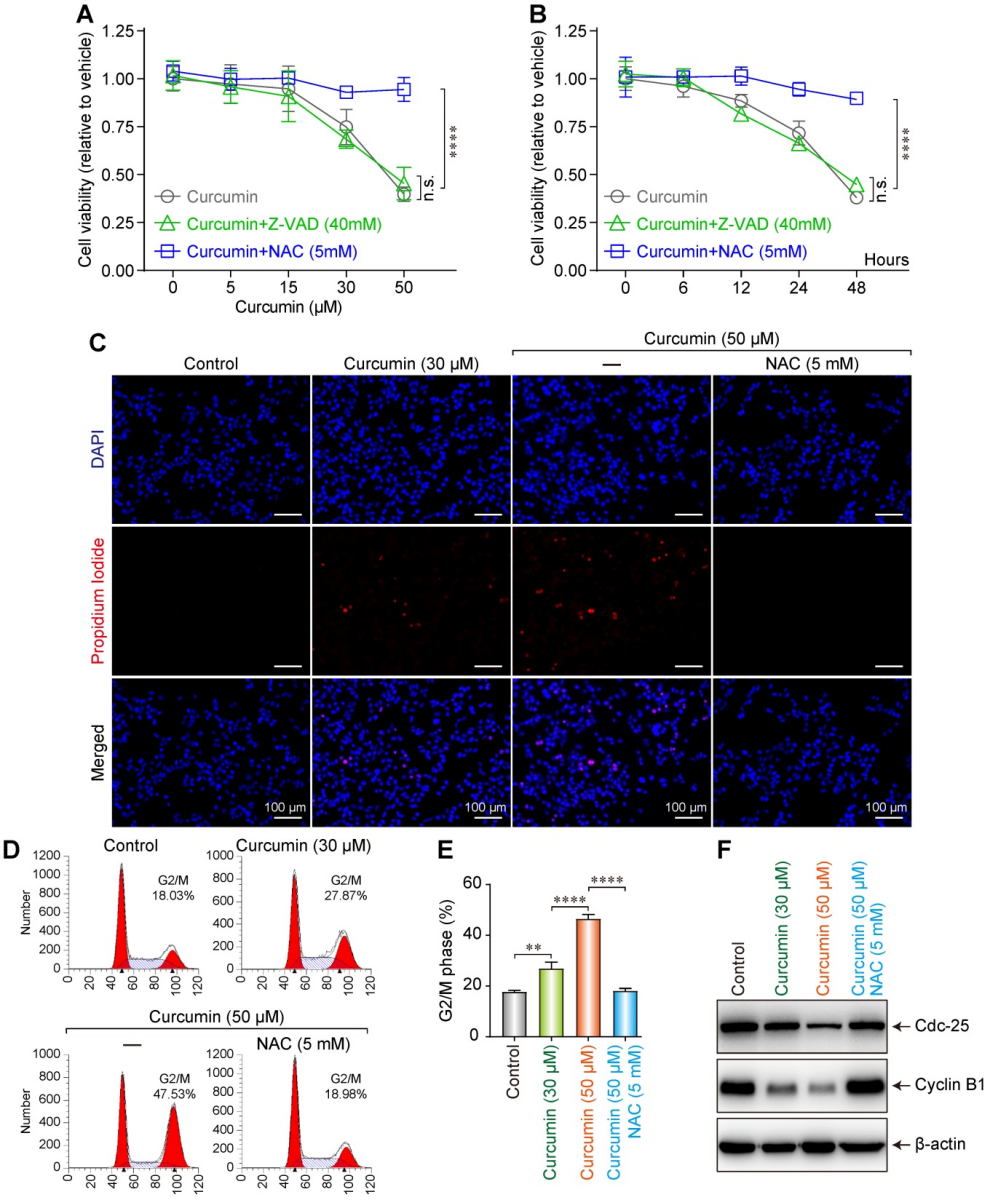
Dose-dependent influence of curcumin on siha cells. **(A)** Viability of siha cells treated with the indicated concentrations of curcumin for 24 h measured by CCK-8 assay. **(B)** Cells treated with 40 µM curcumin for 0,6,12,24,48Hours. Data are calculated as the percentage of control and represent mean (n=3). **(C)** Siha cells treated with curcumin (or with NAC), stained with PI and DAPI, observed under a fluorescent microscope.** (D)** Cell cycle analysis of curcumin-treated cells for 24 h, concentrations as indicated. **(E).** Percentage of cells in each phase of the cell cycle: G1, S and G2/M were estimated after analysis using the modfit software and percentage of cells in G2/M fraction was measured using the CellQuest software. Graphs represent mean±SD (n=3 ). **(F)** Level of cdc25, cyclinB1 proteins estimated by western blotting. Whole-cell extracts were prepared 24h after treatment with different concentrations of curcumin (or with NAC). *β*-actin was used as a loading control.

The cell survival rate demonstrated a decreasing trend, after an initial slight increase, with increasing curcumin concentration. The cell proliferation rate considerably decreased with 50 µmol/L of curcumin. In the cells treated with curcumin + the anti-apoptotic agent Z-VAD 40mM, the survival rate significantly decreased and demonstrated a curcumin dose dependence, whereas the survival rate of the cells treated with curcumin + the antioxidant NAC 5mM did not decrease (Figure 2A). This finding suggests that curcumin exhibits a significant inhibitory effect on the proliferation of SiHa cells in a concentration-dependent manner. Furthermore, this effect can be blocked by NAC but not Z-VAD.

We next evaluated the effect of treatment duration on the survival of SiHa cells. Cells were treated with 40 µmol/L of curcumin and collected after 0, 6, 12, 24, or 48h. We observed that the cell proliferation rate decreased with time (Figure 2B). This finding indicates that curcumin at a concentration of 40 µmol/L can inhibit the proliferation of SiHa cells.

To evaluate the effect of curcumin on the apoptosis of SiHa cells, we treated the cells with 30 or 50 µmol/L of curcumin and then co-stained with 4',6-diamidino-2-phenylindole (DAPI) and propidium lodide (PI). Apoptosis was evaluated under a microscope. Although the control cells remained spindle-shaped without any obvious morphological change, those treated with 30 µmol/L of curcumin featured a polygonal shape. Furthermore, unlike in control (red nuclei not seen), 10% of the nuclei in the curcumin-treated sample stained red, indicating apoptosis (Figure 2C). When the cells were treated with 50 µmol/L of curcumin for 24 h, they became circular and contained lysed nuclei and vacuolated cytoplasm. Of the intact nuclei, approximately 20% stained red. The effects of 50 µmol/L of curcumin were suppressed when the cells were co-treated with 5mM NAC.

Moreover, it was revealed by flow cytometry that curcumin induced G2/M arrest in SiHa cells in a dose-dependent manner. The proportion of the arrested cells was 28.87% when 30 µmol/L of curcumin was used, and this rate became 47.53% when the concentration was increased to 50 µmol/L, while with Cur 50µmol/L+NAC 5mM, the proportion was 18.98% (Figure 2D,E). Cells express different cyclins during the different stages of the cell cycle. At the G2 phase, cyclin B is predominantly expressed. Cyclin B complexes with cyclin-dependent kinases-1 (CDK1) to exert functions related to cell maturation and mitosis. Phosphorylation of the Tyr15/Thr14 sites on CDK1 by Wee1 kinases and Myelin transcription factor 1 (Myt1) kinases inhibits CDK1 activity. Dephosphorylation of these sites by Cdc25 re-activates CDK1, thereby ensuring the continuity of the cell cycle 15,16. Western blot results showed that curcumin treatment downregulated the G2/M-related cyclins B1 and cdc25 in a dose-dependent manner in accordance with the observation of cell cycle arrest (Figure 2F). The G2/M cycle arrest caused by 50 µmol/L curcumin was suppressed by 5mM NAC.

### Curcumin caused apoptosis in siha cells

**Figure 3:**
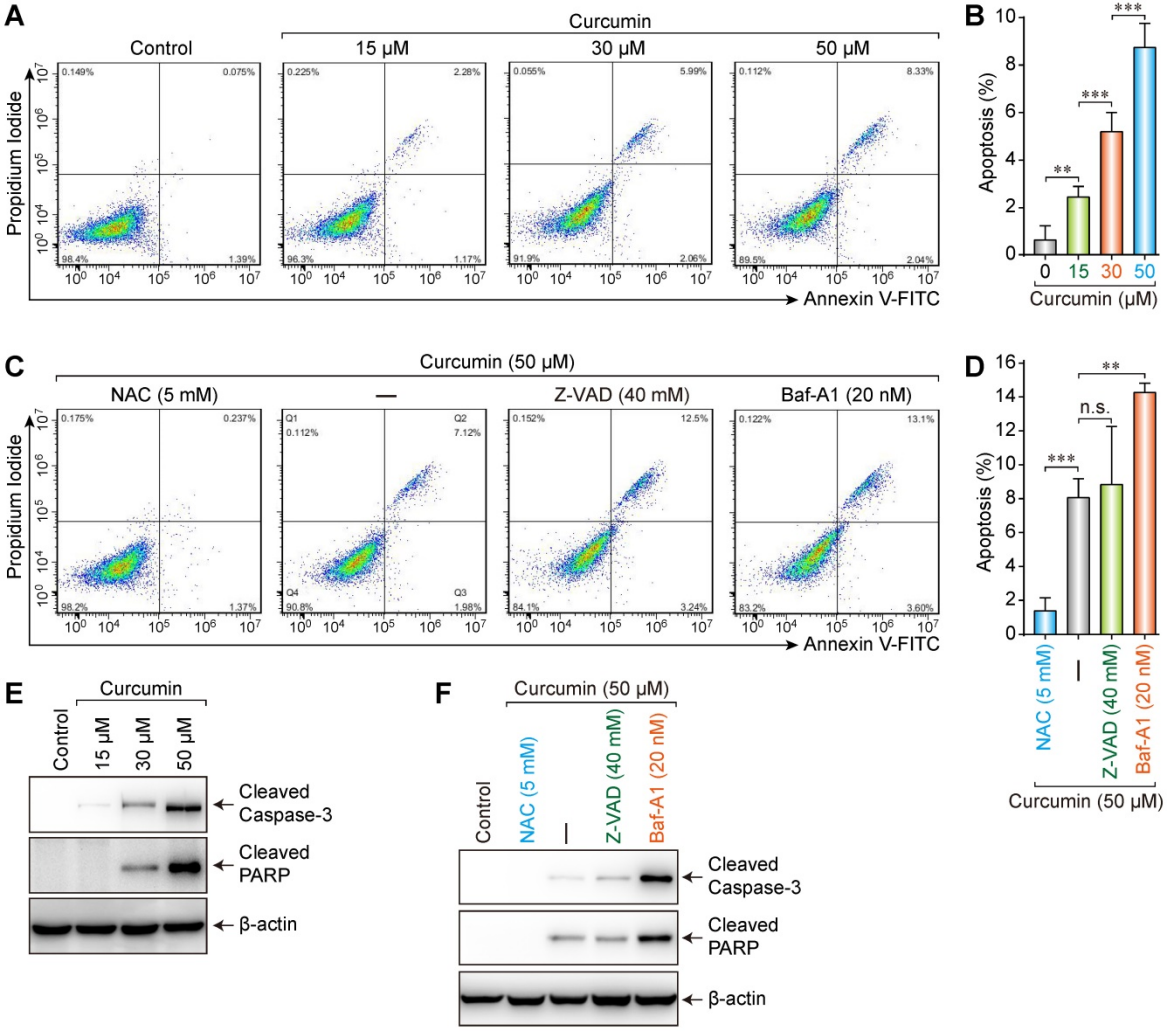
Increased apoptosis was induced by curcumin. **(A)** A caspase assay was used to identify apoptotic cells after 24h treatment with 0,15,30,50 µM curcumin.** (B)** Significant differences were noted in all comparisons: control versus Cur at 15, 30, 50 µM (*p* < 0.001). **(C)** Assay was used to identify apoptotic cells after treatment with curcumin 50 µM or combination treatment of NAC, ZVAD, Baf-A1. **(D)** There were a statistically significant reduction in apoptotic cells in curcumin with NAC (*p* < 0.001) and an increase apoptotic cells with Baf-A1 (*p* < 0.01), while no significant difference was found between cells treated with Cur alone versus Cur combination with ZVAD; **(E)** Western blot analysis of siha cells treated with curcumin in a concentration-dependent manner, for proteolytically cleaved caspase3, cleaved PARP;** (F)** Combination-treated cells (Cur + NAC 5 mM) (Cur+ZVAD 40 mM) (Cur+Baf-A1 20 nM), few active caspase-3 and cleaved caspase 3 were detected in control or (Cur+NAC 5 mM) treated cells. Cells treated with (Cur + Baf-A1 20 nM) had the highest expression of apoptosis markers.

Media with different concentrations (0, 15, 30, or 50 µmol/L) of curcumin were used to treat cells. The results showed that the apoptosis rate increased (0.075%, 2.26%, 5.99%, and 8.33%, respectively) with increasing curcumin concentration (Figure 3A,B). Accordingly, we treated the cells with 50 µmol/L of curcumin with or without 5 mM NAC, 40 mM Z-VAD, or 20 nM Bafilomycin-A1 (Baf-A1) (autophagy inhibitor) for 24 h. NAC could considerably suppress the curcumin-induced apoptosis (0.237%), whereas neither Z-VAD (12.5%) nor Baf-A1 (13.1%) demonstrated a significant suppressive effect (Figure 3C,D). Furthermore, the apoptosis rate in the presence of Baf-A1 further increased compared to that of the curcumin-only group (*p* < 0.01), indicating that inhibiting autophagy increased the apoptosis rate.

Caspase-3 is a key executive molecule in the apoptosis process. It exists as a zymogen in the cytoplasm and is hydrolyzed into 17 kD and 12 kD fragments when activated 17. The 17-kD fragment was detected in SiHa cells treated with curcumin. This finding indicates that curcumin can activate caspase-3, which hydrolyzes poly ADP-ribose polymerase (PARP) into 24 kD and 89 kD fragments, whereby PARP becomes inactivated. Cleaved PARP is a hydrolysate of activated caspase-3 in apoptotic cells and thus a marker of apoptosis.

Western blot results showed that, compared to those in the control group, the expressions of apoptosis-related proteins cleaved Caspase-3 and cleaved PARP increased with 15, 30, or 50 µmol/L of curcumin in a concentration-dependent manner (Figure 3E). Furthermore, whereas apoptosis-related proteins were not detectable in control or cells co-treated with NAC and curcumin, low levels of these proteins were detected in the cells co-treated with Z-VAD and curcumin, and high levels were detected when Baf-A1 was co-administered with curcumin (Figure 3F). These results were consistent with the flow cytometry results.

### Curcumin induced autophagy and autophagy flux in Siha cells

To investigate the effect of curcumin on the autophagy in SiHa cells, double-fluorescently labeled LC3 molecules (Cherry-EGFP-LC3) were used to assess the autophagy and autophagy flux. In cytoplasmic and non-lysosomal vesicles, Cherry-EGFP-LC3 molecules simultaneously emit green and red fluorescence, making the vesicles appear yellow. Alternatively, when autophagosomes and lysosomes fuse into autophagolysosomes, the resultant acidic environment changes the conformation of the EGFP protein and quenches the green fluorescence. Consequently, the double-fluorescent Cherry-EGFP-LC3 molecules only emit red fluorescence, indicating an intact autophagy flux in the cell 18.

**Figure 4:**
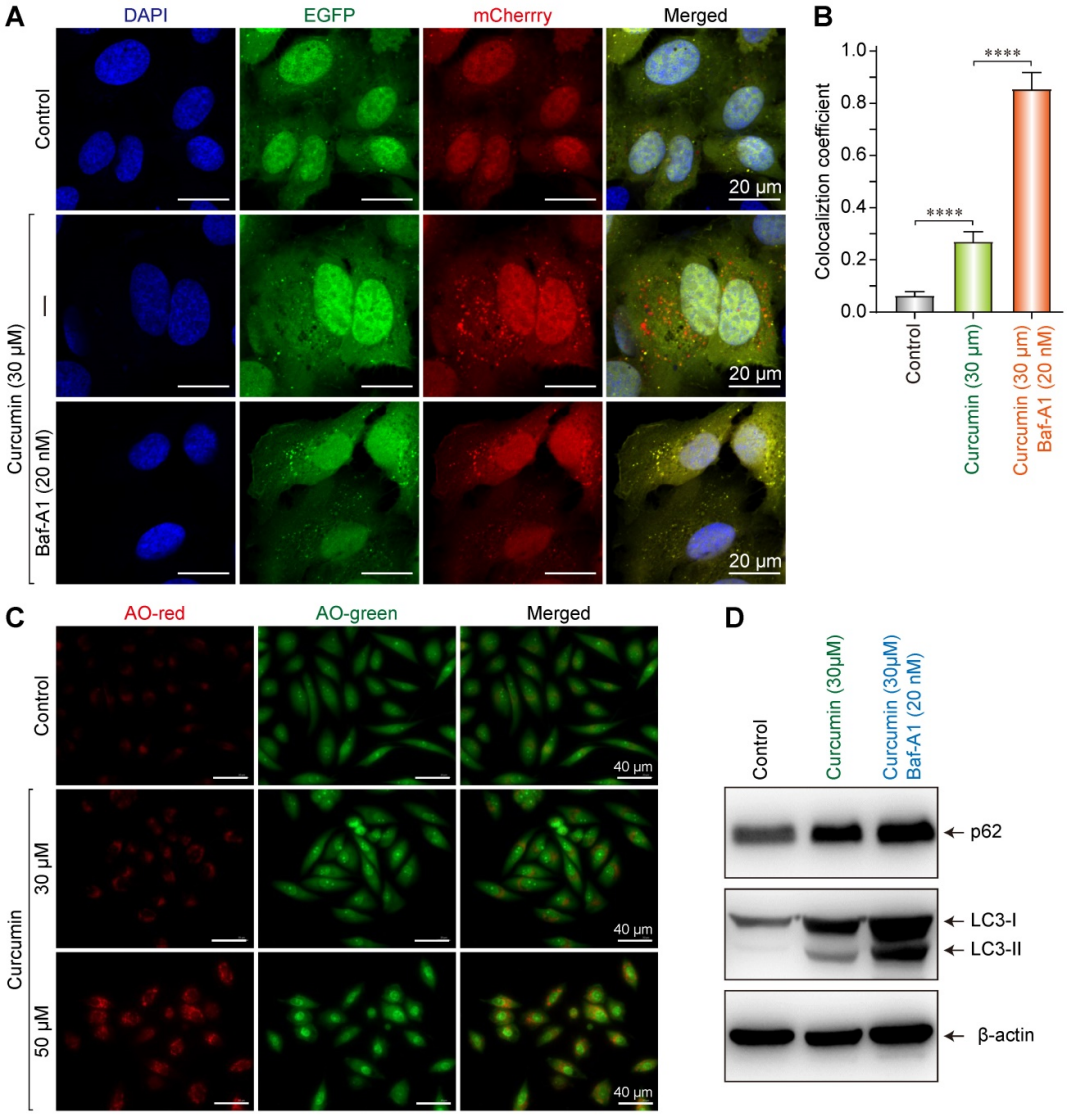
Curcumin induces autophagy in siha cells. **(A)** Confocal images of representative images of EGFP and mCherry fluorescent puncta in siha cells transfected with mCherry-EGFP-LC3 for 24 h, in cells treated with curcumin 30 µM or combination with Baf-A1 (20 nM), autophagic flux was blocked, and more red fluorescent puncta were observed. **(B)** Quantification of EGFP/mCherry colocation coefficient puncta in control or cells treated with Cur only or combination with Baf-A1. **(C)** Fluorescent microscope of acridine orange (AO)-stained vesicles in cells treated with Cur (0, 30, 50 µM) in the section showing cells treated with 50 µM curcumin, there were massive accumulation of AO-positive big vesicles with an acidic content that are remisniscent of autophagosomes.** (D)** Western blot analysis of the conversion of LC3-I to LC3-II, and p62 in cells treated for 24 h with Cur (30 µM) only or combination with Baf-A1 (20 nM), SQSTM1 protein levels in cell lysates were determined by western blot analysis. Equal amounts of proteins were loaded and immunoblot of GAPDH was used as the loading control. The data shown are representative of three independent experiments.

The result showed that in the negative control, only a small number of yellow vesicles were observed due to the basic autophagy of SiHa cells. When SiHa cells were treated with 30 µmol/L of curcumin for 24 h, the number of intracellular fluorescent autophagic vesicles considerably increased. In addition, there were overlaps of red and green fluorescent vesicles, with a larger number of red ones. This result indicates that 30 µM of curcumin can induce autophagy and autophagy flux (Figure 4A), since the cherry fluorescent protein can resist the acidic environment of the lysosome, thereby emitting red fluorescence. After co-administering the autophagy inhibitor Baf-A1 with 30 µmol/L of curcumin, a large number of yellow vesicles were detected in SiHa cells (with statistically significant differences). Since Baf-A1 can block the fusion of autophagy and lysosomes and consequently inhibit the production of autophagolysosomes, this result indicated that under the simultaneous action of curcumin and Baf-A1, autophagy, but not the intact autophagy flux, was activated in SiHa cells (Figure 4A,B). Therefore, as the Cherry-EGFP-LC3 molecules concurrently emitted both red and green fluorescence in autophagic vesicles, multiple yellow vesicles were observed.

After treated with curcumin, cells were then stained with acridine orange to evaluate the formation of intracellular acidic vesicles. As a fluorescent dye, acridine orange emits red fluorescence under the action of acidic hydrolase in autophagolysosome, which changes to a yellow-green fluorescence when combined with DNA. Fluorescence microscopy showed that in cells treated without curcumin, there were few red vesicles as control. Furthermore, in the sample treated with 30 µM of curcumin, the number of red vesicles increased, and some cells demonstrated morphological changes and became oval-shaped. Lastly, in the cells treated with 50 µM of curcumin, a large number of red vesicles were detected (Figure 4C); some cells demonstrated morphological changes and became oval-shaped; and additionally, some cells were vacuolated. These findings indicate that with increasing concentration of curcumin, the autophagy in SiHa cells increases.

Western blot results suggested that 30 µmol/L of curcumin upregulated p62, LC3I, and LC3II in SiHa cells, indicating that autophagy was induced. When 30 µmol/L of curcumin was co-administered with Baf-A1, the accumulation of p62 was more substantial, and the expression of LC3II increased, suggesting that by blocking the autophagy flux, Baf-A1 reduced the degradation of autophagolysosome p62 and LC3II, thereby causing the accumulation of p62 and LC3II (Figure 4D).

### Influence of curcumin on cellular senescence

In normal human cells, inactivation of the p53 and p21 genes can prolong the replicative lives of cells. P53 can mediate cell response to DNA damage, replicative aging due to telomere shortening, as well as stress-induced premature senescence. Its downstream factor p21 can inhibit CDK2 and CDK4, rendering them unable to phosphorylate pRB 19,20. The resultant hypo-phosphorylated pRB can bind to E2F and prevent it from binding to target genes, thereby prohibiting cells from entering the S phase. Accordingly, irreversible cell cycle arrest is triggered, which promotes cellular senescence.

**Figure 5:**
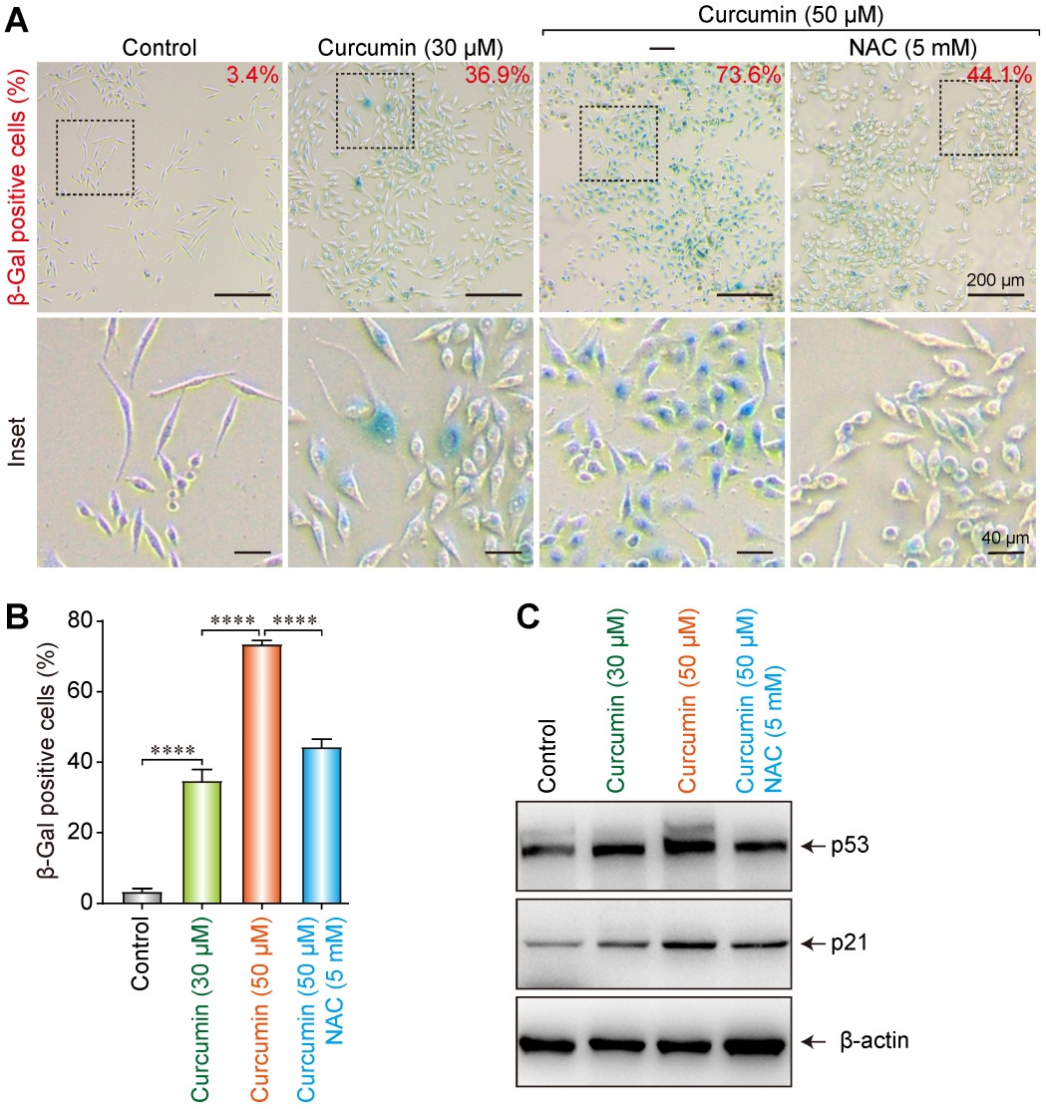
Induction of senescence of the siha cells upon treatment with curcumin.** (A)** Activation of SA-β-gal in curcumin-treated cells at 24 h; Percentage of SA-β-gal positive cells that represent mean (n=3) are given on each image. Bar 200 µm. **(B)** Diagram shows percentage of cells with activation of SA-β-gal in curcumin-treated cells. **(C)** Level of p53 and p21 proteins in cells undergoing senescence upon curcumin treatment. Whole-cell extracts were prepared at indicated time points. β-Actin was used as a loading control.

A cellular senescence β-galactosidase staining kit was utilized to analyze the effect of curcumin on the senescence of SiHa cells. At 30 and 50 µmol/L of curcumin, 36.9% and 73.6% of the cells, respectively, showed an aging phenotype under the microscope. Co-administration of 5 mM of NAC could partially suppress the cellular senescence induced by 50 µmol/L of curcumin, as only 44.1% of the cells in this group showed aging (Figure 5A,B). These findings indicate that curcumin-induced cellular senescence is related to cellular oxidative stress and ROS accumulation. NAC can help to clear intracellular ROS, thereby delaying the cellular senescence process.

To further explore the effect of curcumin on the senescence of SiHa cells, 30 or 50 µmol/L of curcumin was administered to them for 24 h. A control group and a NAC group were also established. Western blot results showed that both 30 and 50 µmol/L of curcumin upregulated the cellular senescence-related proteins p53 and p21 (Figure 5C). However, co-administration of NAC suppressed this increase, suggesting that curcumin induced cellular senescence via the p53-p21 pathway.

## Discussion

ROS generally refer to active compounds containing oxygen produced by organisms during aerobic metabolism. An appropriate amount of ROS can serve as signaling molecules and participate in signal transduction pathways to regulate cell growth, differentiation, and survival, and to engage in inflammatory and immune responses 21. However, excessive ROS levels cause oxidative stress and introduce oxidative damage to important intracellular biomolecules. Such damages impair the functions of DNA, lipids, and proteins, and in turn, the structural and functional integrity of the cell. Ultimately these damages decelerate cell metabolism and activity and can even lead to cell death 22.

An increase in ROS levels can also trigger autophagy. There are two aspects to this process. On the one hand, as the intracellular ROS accumulate, some critical organelles are destroyed, impairing cellular functions and thus inducing pathological changes in organs 23. Autophagy can enable cells to survive under such circumstances by clearing the structures impaired by ROS. On the other hand, autophagy increases the intracellular ROS levels and thus forms a positive feedback loop, increasing the oxidative damages and ultimately causing autophagic cell death 24.

Autophagy is a self-protection mechanism of cells under stress. It is regulated and induced by multiple factors, among which the ROS-JNK pathway is one of the most important 25. Once activated by ROS, JNK1 can directly phosphorylate Bcl-2 protein and dissociate it from Beclin 1, thereby initiating autophagy 26,27. In addition, the ROS-JNK signaling pathway can induce autophagy by directly upregulating the key autophagy genes autophagy gene 7 (ATG7) and ATG5 through a Beclin1-independent mechanism 28.

Our study has found that curcumin can inhibit cell proliferation, the effect of which is both dose- and time-dependent. When treated with 50 µM of curcumin for 24 h, approximately 40% of SiHa cells survived. Co-staining of the treated cells with PI and DAPI suggested 20% of the cells underwent apoptosis. These results indicated a considerable proportion of the cells underwent apoptosis-independent cell death. Further examination of the intracellular autophagy level showed that the curcumin treatment promoted the transformation of microtubule-associated protein1 light chain3 as well as the degradation of autophagosome markers, such as sequestome-1 (SQSTM1). These results demonstrate that curcumin can induce both autophagy and intact autophagy flux, while NAC can block this process, indicating that the generation of ROS plays a crucial role in inducing autophagy.

Studies have revealed that H_2_O_2_ produced in mitochondria can affect the activity of the cysteine protease Atg4, which is an important factor in the autophagy process. Atg4 can modify Atg8, which is essential for the formation of autophagic vesicles 29,30, suggesting that the accumulation of ROS can induce autophagy. Our study discovered that curcumin induced autophagy and ROS accumulation in SiHa cells. The co-administration of the antioxidant NAS substantially suppressed the increase in the intracellular ROS levels and the degree of autophagy, whereas the co-administration of the autophagy inhibitor increased the intracellular ROS levels. These findings suggest that there is a restrictive mutual role between autophagy and ROS accumulation during this process; the generation of ROS promotes autophagy, which in turn clears the intracellular ROS, thereby maintaining cell homeostasis.

Apoptosis and autophagy are two important cell functions that are closely related 31. A moderate level of ROS is sufficient to cause the transient activation of the JNK signaling pathway and to increase autophagy through the Beclin1 pathway, but not sufficient to cause apoptosis. However, excessive ROS would continuously activate the JNK pathway, thereby causing mitochondrial-pathway-mediated apoptosis 32. ROS activate JNK through the bispecific kinase JNKK, while the activated JNK can then upregulate pro-apoptotic proteins, such as p53, Bax, Fasl, and TNF through the transcription factor AP-1. These pro-apoptotic factors ultimately activate Caspase 3 and induce apoptosis 33,34. In this study, 50 µM of curcumin substantially induced apoptosis in SiHa cells. Additionally, the apoptosis-related factors cleaved caspase-3 and cleaved PARP were upregulated. Co-administration of the antioxidant NAC suppressed the increase in the apoptosis rate, indicating that ROS play an important role in curcumin-induced apoptosis. When the autophagy inhibitor Baf-A1 was administered simultaneously, autophagy was inhibited and the apoptosis rate increased, suggesting that moderate autophagy can protect cells from further apoptosis under stress.

Our results also showed that curcumin induced G2/M arrest in a considerable number of SiHa cells as well as significant changes in G2/M-related cdc25 and cyclinB1 protein levels. In addition, this process could be effectively suppressed by the antioxidant NAC, indicating that ROS play an equally important role in curcumin-induced cell cycle arrest.

Furthermore, dramatic morphological changes of the SiHa cells were observed upon curcumin treatment. For example, the cells became flattened and enlarged, which is a morphological feature of cellular senescence. Cellular senescence refers to declined cell physiological functions, upregulation of senescence proteins, and reduced proliferation 35. It has been revealed that tumor suppressors, such as p53, p16, and Rb, participate in cellular senescence, although the specific underlying mechanism(s) remain unclear 36.

Curcumin has been suggested to induce the nuclear accumulation of forkhead box transcription factor 1 (FOXO1), which in turn upregulates p27, p21, and p130, and inhibits cyclin D1 and D2, thereby further inhibiting cell cycle transition. In addition, curcumin can downregulate survivin, which then leads to abnormal mitosis 37. Although abnormal mitosis does not necessarily cause cell death, abnormal polynuclei and chromosome splitting are common signs of cellular senescence 38.

Our results showed that cells treated with curcumin had an increased amount of aging-related factors. However, co-administration of the antioxidant NAC suppressed their accumulation, in line with the conclusion that ROS play a crucial role in curcumin-induced cellular senescence 39. Under normal circumstances, the relatively stable intracellular p53 protein activates the downstream transcription factor p21. Alternatively, under cellular senescence, Rb enters a phosphorylated state and binds to E2F family proteins to inhibit their transcription, thereby affecting both the cycle and physiological activities of cells. Our study further discovered that curcumin upregulated p53 and p21 and the number of SA-β-gal positive cells in the SiHa cell line. Overexpression of the senescence marker p21 has been suggested to induce the senescence of cancer cells. Therefore, it is possible that curcumin induces the senescence of SiHa cells through the p53-p21 pathway. Cellular senescence has previously been attributed to normal cells reaching the limit of proliferation (the so-called replicative senescence). However, recent clinical trials have indicated that cellular senescence is a common response of tumor cells under anticancer drugs or chemotherapy 40.

Although senescent cells are still viable and metabolically active, they can effectively restrict tumor growth and even facilitate the elimination of tumor cells by activating the innate immune response. As tumor cells feature uncontrolled proliferation, the induction of cell cycle arrest can be adopted as a method to inhibit tumor cell proliferation and to further trigger cellular senescence, providing a new strategy for cancer treatment 41.

The potential antitumor effect of curcumin has attracted much attention in the cancer field. Curcumin can kill tumor cells by regulating various signaling pathways. Although the role of curcumin differs among different cell lines, it is clear that curcumin, as a drug capable of inducing cell death, can become an effective tumor-suppressive tool 42. In summary, curcumin can regulate the intracellular ROS levels, induce autophagy and apoptosis, trigger G2/M cell cycle arrest, and mediate the senescence of cells trough the p53-p21 pathway. Our study has provided a solid theoretical basis for the clinical treatment of cervical cancer, although further studies are required on the details of the underlying molecular mechanisms.
